# Misinformation and herd behavior in media markets: A cross-national investigation of how tabloids’ attention to misinformation drives broadsheets’ attention to misinformation in political and business journalism

**DOI:** 10.1371/journal.pone.0241389

**Published:** 2020-11-11

**Authors:** Bartosz Wilczek

**Affiliations:** Faculty of Communication, Culture and Society, Università della Svizzera Italiana, Lugano, Switzerland; Beihang University, CHINA

## Abstract

This study develops and tests a theoretical framework, which draws on herd behavior literature and explains how and under what conditions tabloids’ attention to misinformation drives broadsheets’ attention to misinformation. More specifically, the study analyzes all cases of political and business misinformation in Switzerland and the U.K. between 2002 and 2018, which are selected based on corresponding Swiss and U.K. press councils’ rulings (N = 114). The findings show that during amplifying events (i.e., election campaigns and economic downturns) tabloids allocate more attention to political and business misinformation, which, in turn, drives broadsheets to allocate more attention to the misinformation as well–and especially if the misinformation serves broadsheets’ ideological goals. Moreover, the findings show differences between Swiss and U.K. media markets only in the case of business misinformation and suggest that the attention allocation process depends in particular on the strength of the amplifying event in a media market. Thereby, this study contributes to the understanding of how and under what conditions misinformation spreads in media markets.

## Introduction

The spread of inaccurate information has become a serious concern around the world. After all, the functioning of democracies and economies relies on well-informed publics [1–5]. The spread of unverified content, however, creates the risk that political and economic outcomes “will rest on misinformation” ([6] page 736).

More specifically, “[s]ome misinformation is simply erroneous information or containing factual errors due to unintentional or innocent mistakes. But some misinformation is false information intentionally created to mislead and misinform people with an agenda” ([7] page 2). Inaccurate information, which is intentionally produced and spread to harm, is called disinformation [8] or fake news [9]. However, as Ha, Perez and Ray rightly argue, “the intention of the message is difficult to be ascertained by the receiver who may be the subsequent propagator of the message” ([7] page 2). Accordingly, “misinformation is an appropriate descriptor of false information until it is confirmed as disinformation”.

In fact, democracies and economies will be even more at risk if misinformation is not only spread by tabloids (i.e., newspapers with a mass- or mid-market orientation) but also by broadsheets (i.e., newspapers with an up-market orientation) [10]. After all, as “bouncers of the public sphere and truth’s keeper[s]” ([11] page xi), broadsheets are considered to be particularly relevant for the functioning of societies [12].

Moreover, the “leading thought” in communication science has been that “news flows from elite news media […] to other media outlets” ([13] page 181). More specifically, previous literature has argued that broadsheets are particularly important opinion leaders among traditional news media and set the news agenda of other types of news media (e.g., [14–18]). Sutter, in turn, argues that during events, which increase news media’s attention to issues (e.g., [19]), “reporting standards” of tabloids will “drive coverage”, i.e., they will spill over to broadsheets, which eventually will result in “lowest common denominator journalism” ([20] page 747). This suggests that, under specific conditions, tabloids’ attention to misinformation will drive broadsheets’ attention to misinformation, which will cause “greater harm” than had specific issues “been relegated to the tabloids” ([20] page 747).

However, so far, this relationship has not been investigated. While previous research has focused on the spread of misinformation on social media (e.g., [1, 21–26], the diffusion of misinformation in news media has found less attention and is, therefore, less well understood [7]. Silverman [27] analyzed how rumors–i.e., claims of factual nature that are not yet determined to be true or false–circulated on international news websites between August and December 2014. Vargo, Guo and Amazeen [28] investigated the agenda-setting power of “fake news” websites on fact-based news websites in the U.S. between 2014 and 2016. Guo and Vargo [13], in turn, analyzed this association in the U.S. between September and November 2016 and compared different types of “fake news”. Accordingly, how interactions between tabloids and broadsheets play out in the spread of misinformation under different conditions remains unclear.

Therefore, this study develops and tests a theoretical framework, which draws on herd behavior literature and explains how and under what conditions tabloids’ attention to misinformation drives broadsheets’ attention to misinformation. More specifically, the study applies a replication logic and investigates this relationship in two news ecosystems, i.e., in political and business journalism. For that purpose, the study analyzes all cases of political and business misinformation in Switzerland and the U.K. between 2002 and 2018, which are selected based on corresponding Swiss and U.K. press councils’ rulings (N = 114).

Herd behavior literature is particularly useful to investigate interactions between tabloids and broadsheets regarding the allocation of attention to misinformation because it consists of a broad set of concepts, which offer differentiated explanations for imitation in media markets. Moreover, the study investigates the spread of misinformation in political and business journalism because political and economic systems are cornerstones of societies where misinformation might cause particularly great damage (e.g., [3, 6]). Finally, the study compares Swiss and U.K. media markets because they differ in terms of regulation and structure [10], which might affect the allocation of attention to misinformation.

## Theoretical framework

**Fig 1:**
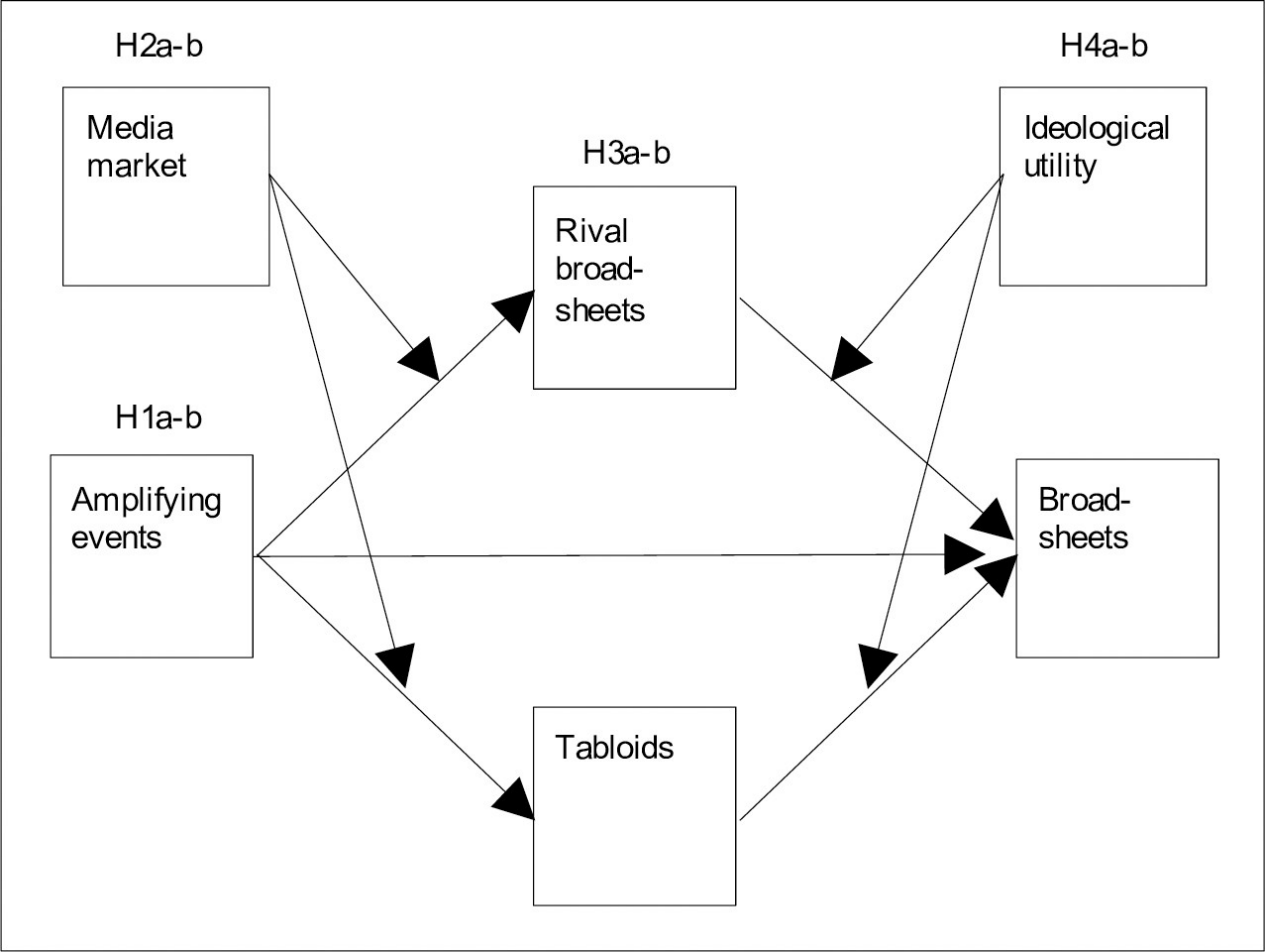
Theoretical framework.

Fig 1 presents the theoretical framework of this study, which draws on herd behavior literature and explains how and under what conditions tabloids’ attention to misinformation drives broadsheets’ attention to misinformation. In the following, the theoretical building blocks are discussed, and the corresponding hypotheses are developed.

Herd behavior refers to imitation between actors and has several preconditions [29]. First, obviously, more than one actor needs to be involved. Second, the situation must have an iterative character with sequential decision-making of the actors involved. Third, actors must be able to observe each other’s actions [30]. All these conditions apply to media markets [31, 32] where broadsheets can monitor how tabloids and rival broadsheets allocate their attention in news reporting and, based on this monitoring, decide whether to imitate tabloids and rival broadsheets and, thus, to allocate attention to misinformation as well.

The attention allocation process (Fig 1) starts when political or business misinformation enters a media market, i.e., when a first-mover news outlet publishes political or business misinformation. In such a situation, tabloids and rival broadsheets will have to decide whether to imitate the first-mover news outlet, i.e., whether to allocate attention to the misinformation. In fact, it is argued that tabloids and rival broadsheets will allocate more attention to misinformation if it breaks during amplifying events [33] because such events will increase competition among news media in news reporting [31]. Higher competition, in turn, will increase news media’s incentives to allocate attention to information–even if its accuracy is uncertain. After all, as “[t]ime pressure increases” the more news media cover an issue, “[c]ompetition for scoops, or to avoid being scooped, can lead to reporting without sufficient confirmation” ([20] page 747).

More specifically, it is expected that national elections and economic downturns will represent such amplifying events in political and business journalism, respectively. In fact, news media will allocate more attention to political misinformation during later stages of election campaigns. After all, the closer election dates approach, the more will news media compete to shape election outcomes [28, 34]. Moreover, news media will allocate more attention to business misinformation during stronger economic downturns. After all, the more pronounced economic crises are, the more will news media compete to defend their economic positions, for instance, to advance or prevent market regulation [35, 36].

However, it is argued that tabloids will allocate more attention to political and business misinformation during election campaigns and economic downturns, respectively, than rival broadsheets [20]. As tabloids face pressures of mass- or mid-markets, they will invest fewer resources for verifying information and they will more likely adopt information in situations in which accuracy is uncertain than broadsheets, which have an up-market orientation [10, 37]. This leads to the following hypotheses:

**H1a**: Tabloids allocate more attention to political misinformation the closer campaigns approach election dates than rival broadsheets.
**H1b**: Tabloids allocate more attention to business misinformation the stronger economic downturns are than rival broadsheets.

Moreover, actors’ decisions to engage in imitation are (often) based on cost-benefit analyses [38]. Accordingly, it is expected that tabloids’ and rival broadsheets’ susceptibility to imitate the first-mover news outlet and, thus, to allocate attention to political and business misinformation will depend on the media market, which will shape news media’s incentives through its regulation and structure [10].

More specifically, press councils regulate news media by defining journalistic accuracy standards and by sanctioning news media, which publish inaccurate information [39]. However, depending on the media market, press councils have different sanctioning mechanisms at their disposal [40, 41]. The Swiss press council [42] has been only able to publicly communicate its rulings and, thereby, make accuracy violations transparent. The U.K. press councils *Press Complaints Commission* [43] and *Independent Press Standards Organization* [44], in turn, have been also able to force accuracy violators to publish corrections and adjudications. This might–over time–decrease audiences’ trust and ultimately news media’s financial performance [45, 46]. Accordingly, higher expected costs due to stronger press councils’ sanctions might provide stronger incentives to verify information and, thus, might constrain news media to allocate attention to misinformation.

However, the Swiss press council has operated in a smaller media market, which has consisted of fewer tabloids and broadsheets and, therefore, has been characterized by lower competition [10, 40, 41]. The U.K. press councils, in turn, have operated in a larger media market, which has consisted of more tabloids and broadsheets and, therefore, has been characterized by higher competition [10, 40, 41]. As discussed above, higher competition increases news media’s incentives to allocate attention to information–also in situations in which accuracy is uncertain [20]. Accordingly, it is assumed that higher expected benefits due to stronger competition in a media market will outweigh higher expected costs due to stronger press councils’ sanctions and, thereby, will drive news media to allocate attention to misinformation. This leads to the following hypotheses:

**H2a:** The media market moderates the relationships between election campaigns as well as tabloids’ and rival broadsheets’ attention to political misinformation, i.e., higher competition (U.K.) leads to more attention.
**H2b:** The media market moderates the relationships between economic downturns as well as tabloids’ and rival broadsheets’ attention to business misinformation, i.e., higher competition (U.K.) leads to more attention.

The attention allocation process (Fig 1) continues with broadsheets’ decisions whether to imitate tabloids and rival broadsheets, i.e., whether to allocate attention to political or business misinformation as well. It is expected that the more attention tabloids and rival broadsheets allocate to political and business misinformation, the more attention will broadsheets allocate to the misinformation too. Specific incentives will drive such imitation.

Scharfstein and Stein [47] who introduced reputational cascades argue that actors engage in imitation when they are uncertain about a choice and if they risk jeopardizing their reputation due to an adverse decision. By imitating other actors, they aim to maintain social approval. After all, if a decision should turn out to be wrong, they will be able to “share the blame” ([47] page 466). In line with this argument, Hamilton states that as “the number of [news media] […] covering a story grows, an individual [news outlet] […] may be more likely to simply go with the angle and events developed by previous [news media] […]” to avoid sanctions for “going against a perceived wisdom in coverage” ([48] pages 22–23).

Bikhchandani, Hirshleifer and Welch [49], in turn, introduced informational cascades and argue that actors engage in imitation if obtaining information is costly. More specifically, actors “will buy information […] only up to the point where the information yields no more net benefits than just following signals emitted by others” ([50] page 16). This holds also for news media. After all, they will save costs of news production if they adopt previously published information. “Rather than investigate and develop a story, a [news outlet] […] may look at the efforts of others and use a similar take on a news event” ([48] page 28).

Moreover, Kuran and Sunstein [6] who introduced availability cascades argue that actors use different heuristics to evaluate their environment. The availability heuristic “involves estimating the probability of an event on the basis of how easily instances of it can be brought to mind” ([6] page 706). During availability cascades, actors imitate others because they take the simple availability of information as an indication of its reliability and relevance. Such availability cascades occur also in media markets. After all, according to Kuran and Sunstein, a “typical newspaper […] incurs a large penalty whenever it falls behind its rivals in reporting ‘breaking news’” ([6] page 750). Consequently, once “a development breaks, other news organizations must respond as the new development itself becomes news; followers report the development because everybody else is reporting it” ([20] page 747).

In fact, decision-makers are more susceptible to join reputational and informational cascades if they consist of actors who are expected to have (more) reliable information [50, 51]. This suggests that broadsheets will imitate rival broadsheets in particular. However, in the case of availability cascades, the perceived availability of information is the underlying driver of imitation [6]. This, in turn, indicates that broadsheets will imitate tabloids in particular. After all, as discussed above, tabloids will allocate more attention to political and business misinformation than rival broadsheets. Thereby, they will make the misinformation more publicly available and, therefore, put more pressure on broadsheets to allocate attention to the misinformation as well. This leads to the following hypotheses:

**H3a**: Broadsheets allocate more attention to political misinformation due to tabloids’ increased attention to the misinformation than due to rival broadsheets’ increased attention to the misinformation.
**H3b:** Broadsheets allocate more attention to business misinformation due to tabloids’ increased attention to the misinformation than due to rival broadsheets’ increased attention to the misinformation.

Finally, as argued above, actors’ decisions to engage in imitation are (often) based on cost-benefit analyses [38]. Accordingly, it is expected that broadsheets’ susceptibility to imitate tabloids and rival broadsheets and, thus, to allocate attention to political and business misinformation will depend on the ideological utility of misinformation, i.e., ideological costs and benefits of the misinformation will shape broadsheets’ incentives [1, 52].

More specifically, broadsheets will incur costs from allocating attention to misinformation if the misinformation contradicts their ideological goals, e.g., if it undermines actors and issues that relate to shared political or economic positions. Accordingly, it is argued that broadsheets will allocate less attention to misinformation, which incurs ideological costs. However, broadsheets will benefit from allocating attention to misinformation if it serves their ideological goals, e.g., if it undermines actors and issues that relate to contrary political or economic positions. For instance, such misinformation will advance broadsheets’ ideological goals by influencing elections [52] and shaping markets [36]. Accordingly, it is argued that broadsheets will allocate more attention to misinformation, which has ideological benefits. This leads to the following hypotheses:

**H4a**: The ideological utility of political misinformation moderates the relationships between tabloids’ and rival broadsheets’ attention to misinformation as well as broadsheets’ attention to misinformation, i.e., ideological benefits lead to more attention.
**H4b**: The ideological utility of business misinformation moderates the relationships between tabloids’ and rival broadsheets’ attention to misinformation as well as broadsheets’ attention to misinformation, i.e., ideological benefits lead to more attention.

## Methods

### Data

The sampling was conducted in four steps. First, two media markets were selected, i.e., Switzerland and the U.K. As discussed above, these media markets were investigated because they differ in terms of regulation and structure [53, 54].

Second, Swiss and U.K. cases of political and business misinformation were sampled. As argued above, the spread of misinformation in political and business journalism was investigated because political and economic systems are cornerstones of societies where misinformation might cause particularly great damage. For that purpose, all rulings were collected in which Swiss and U.K. press councils upheld accuracy violations in political and business journalism that were committed between January 2002 and December 2018 by news outlets located in the German-speaking part of Switzerland and England. This time frame was chosen to increase the sample size (and because articles of all investigated news outlets have been accessible only since 2002). More specifically, all rulings regarding political and business news were considered, which covered national, regional or local issues.

In the U.K., the *Independent Press Standards Organization* replaced the *Press Complaints Commission* in 2014. Accordingly, in the U.K., rulings of both press councils were analyzed. The rulings were retrieved via Swiss and U.K. press councils’ websites, downloaded and saved in the research database. This resulted in overall N = 114 cases of misinformation. More specifically, regarding political misinformation, N = 56 cases were analyzed (Switzerland: N = 15; U.K.: N = 41). Moreover, regarding business misinformation, N = 58 cases were analyzed (Switzerland: N = 32; U.K.: N = 26).

Third, the Swiss and U.K. newspapers were sampled. Drawing on fög [37], in the German-speaking part of Switzerland, the broadsheets *Neue Zürcher Zeitung* and *Tages-Anzeiger* as well as the tabloid *Blick* were selected. In England, the broadsheets *The Daily Telegraph*, *Financial Times*, *The Guardian*, *“i”*, *The Independent* and *The Times* as well as the tabloids *Daily Express*, *Daily Mail*, *Daily Mirror*, *Daily Star* and *The Sun* were selected [10].

Accordingly, in the German-speaking part of Switzerland, all supraregional daily paid newspapers were investigated. This holds also for England–with one exception: *Morning Star* was not considered, as its articles were not accessible for the whole analyzed time frame (however, compared to the investigated newspapers, *Morning Star* has a considerably lower circulation). Moreover, in both countries, free papers were not included in the sample, as their articles were not accessible for the whole investigated time frame either. As indicated below, specific subsamples were used regarding the mediator and dependent variables.

Fourth, all print articles were collected in which the investigated broadsheets and tabloids adopted political and business misinformation during the first three months since the misinformation was first published. Articles were retrieved via Factiva, downloaded and saved in the research database. For each case of misinformation, articles were collected in several steps using different keywords and search strings [55]. The keywords related to the following characteristics of the misinformation: brand of the first-mover news outlet; subject(s) of the misinformation (i.e., actors and issues); claim(s) of the misinformation (i.e., verbatim and paraphrased). The characteristics were determined via press councils’ rulings.

This approach assured that all articles were considered, which covered the misinformation (and that articles were excluded from the analysis, which reported on an issue without adopting the misinformation; however, for the sampling, these articles were also downloaded and saved in the research database). This resulted in N = 184 articles. More specifically, N = 118 political articles (Switzerland: N = 11; U.K.: N = 107) and N = 66 business articles (Switzerland: N = 13; U.K.: N = 53) were analyzed.

For the analysis of broadsheets’ and tabloids’ articles, a codebook was developed and pre-tested [55]. The category system of the codebook incorporated all corresponding variables and measures (see below). Moreover, to assure consistency of coding over time, the data collection was performed twice, i.e., in two consecutive waves [56].

### Measurement

#### Independent variables

In the case of political journalism, the independent variable relates to election campaigns. The proximity to election dates was measured based on an 18-point scale: 1 month before election = 18; 18 months before election = 1. After all, in the investigated countries, pre-election opinion polls were conducted over the course of this time frame. Non-election periods were coded with = 0. The dates of the national parliamentary elections were determined via websites of the Swiss and U.K. parliaments.

As Table 1 shows, in the U.K. (*M* = 4.33; *SD* = 5.627), first-mover news outlets published political misinformation on average closer to the election dates than first-mover news outlets in Switzerland (*M* = 3.72; *SD* = 5.049).

**Table 1:** Descriptive statistics (political journalism).

	Total		CH		U.K.	
	M	SD	M	SD	M	SD
Election campaigns	4.20	5.502	3.72	5.049	4.33	5.627
Media market: U.K. (ref. = CH)	.80	.405	.00	.000	1.00	.000
Attention to misinformation: RB	9.38	24.636	2.95	11.657	11.04	26.770
Attention to misinformation: TA	15.32	26.952	7.21	26.977	17.40	26.668
Ideological utility: IB (ref. = IC)	.58	.496	.59	.501	.58	.497
First mover: HS (ref. = LS)	.35	.477	.17	.384	.39	.490
Misinformation: News value	18.56	4.455	16.14	4.373	19.18	4.279
Misinformation: Year	12.62	4.832	9.00	4.870	13.55	4.381
Broadsheets: RE (ref. = NR)	.82	.388	1.00	.000	.77	.423
Attention to misinformation: BR	3.10	10.284	2.88	11.413	3.16	10.029
	N = 142		N = 29		N = 113	

M = mean; SD = standard deviation; U.K. = English part of the U.K.; CH = German-speaking part of Switzerland; RB = rival broadsheets; TA = tabloids; IB = ideological benefits; IC = ideological costs; HS = higher expert status; LS = lower expert status; RE = regulated by press council; NR = not regulated by press council; BR = broadsheets.

Moreover, in the case of business journalism, the independent variable relates to economic downturns. This was measured based on GDP per capita rates, which are coincident indicators and reflect the current state of an economy, i.e., higher GDP per capita rates indicate economic upturns. For the statistical analysis, the rates were multiplied with -1; therefore, higher values indicate economic downturns. Swiss and U.K. GDP per capita rates were determined via the website of OECD.

As Table 2 shows, first-mover news outlets in the U.K. (*M* = -3.38; *SD* = 1.138) published business misinformation on average during stronger economic downturns than first-mover news outlets in Switzerland (*M* = -4.75; *SD* = 3.614).

**Table 2:** Descriptive statistics (business journalism).

	Total		CH		U.K.	
	M	SD	M	SD	M	SD
Economic downturns	-4.00	2.658	-4.75	3.614	-3.38	1.138
Media market: U.K. (ref. = CH)	.54	.500	.00	.000	1.00	.000
Attention to misinformation: RB	5.18	11.486	1.89	6.166	7.92	13.971
Attention to misinformation: TA	9.55	23.651	1.40	4.395	16.35	30.234
Ideological utility: IB (ref. = IC)	.49	.502	.51	.504	.48	.503
First mover: HS (ref. = LS)	.26	.441	.21	.413	.30	.462
Misinformation: News value	20.26	4.809	18.95	4.322	21.36	4.948
Misinformation: Year	12.14	5.183	9.59	4.978	14.27	4.341
Broadsheets: RE (ref. = NR)	.87	.342	1.00	.000	.75	.434
Attention to misinformation: BR	2.03	6.058	1.77	5.892	2.24	6.226
	N = 134		N = 61		N = 73	

M = mean; SD = standard deviation; U.K. = English part of the U.K.; CH = German-speaking part of Switzerland; RB = rival broadsheets; TA = tabloids; IB = ideological benefits; IC = ideological costs; HS = higher expert status; LS = lower expert status; RE = regulated by press council; NR = not regulated by press council; BR = broadsheets.

#### Dependent variables

The dependent variables relate to the amount of attention, which broadsheets allocated to political and business misinformation that was first published by another news outlet in the respective country. In the German-speaking part of Switzerland, all broadsheets were investigated: *Neue Zürcher Zeitung* and *Tages-Anzeiger*. In England, the investigated sample consisted of the following broadsheets: *The Daily Telegraph*, *The Guardian* and *The Times*. These three broadsheets were selected due to their particularly high circulations and because they have existed during the whole investigated time frame.

Drawing on Lacy, Watson, Riffe and Lovejoy [55] as well as fög [37], the amount of attention to political and business misinformation was measured based on the following items: duration of news coverage (number of days); number of articles; length of articles (sum of all articles: number of words divided by 100); position of inaccurate information in article titles (sum of all articles: 1 = not in the title or lead; 2 = in the lead; 3 = in the title) and article texts (sum of all articles: 1 = in the third part; 2 = in the second part; 3 = in the first part). For each case of political and business misinformation and for all five investigated broadsheets (i.e., *Neue Zürcher Zeitung* and *Tages-Anzeiger* as well as *The Daily Telegraph*, *The Guardian* and *The Times*), the items were summed up into attention indices.

As Table 1 shows, broadsheets in the U.K. (*M* = 3.16; *SD* = 10.029) allocated on average more attention to political misinformation than broadsheets in Switzerland (*M* = 2.88; *SD* = 11.413). This holds also for business misinformation (Table 2): U.K. (*M* = 2.24; *SD* = 6.226); Switzerland (*M* = 1.77; *SD* = 5.892).

#### Mediators

The first set of mediators relates to the amount of attention, which rival broadsheets allocated to political and business misinformation. In the German-speaking part of Switzerland, the following broadsheets were considered: *Neue Zürcher Zeitung* and *Tages-Anzeiger*. In England, the following broadsheets were analyzed: *The Daily Telegraph*, *The Guardian* and *The Times* and also *Financial Times*, *“i”* and *The Independent*.

The second set of mediators relates to the amount of attention, which tabloids allocated to political and business misinformation. In the German-speaking part of Switzerland, the following tabloid was considered: *Blick*. In England, the following tabloids were analyzed: *Daily Express*, *Daily Mail*, *Daily Mirror*, *Daily Star* and *The Sun*.

Rival broadsheets’ and tabloids’ attention to political and business misinformation was measured based on the same items as in the case of the dependent variables: duration of news coverage (number of days); number of articles; length of articles (sum of all articles: number of words divided by 100); position of inaccurate information in article titles (sum of all articles: 1 = not in the title or lead; 2 = in the lead; 3 = in the title) and article texts (sum of all articles: 1 = in the third part; 2 = in the second part; 3 = in the first part). For each case of political and business misinformation and for all five investigated broadsheets (i.e., *Neue Zürcher Zeitung* and *Tages-Anzeiger* as well as *The Daily Telegraph*, *The Guardian* and *The Times*), the items were summed up into two types of attention indices, i.e., regarding the total attention of rival broadsheets and regarding the total attention of tabloids in the respective country. Moreover, the number of rival broadsheets and tabloids, which adopted political or business misinformation, was added to the attention indices.

As Table 1 shows, in the U.K. and in Switzerland, tabloids (U.K.: *M* = 17.40; *SD* = 26.668; CH: *M* = 7.21; *SD* = 26.977) allocated more attention to political misinformation than rival broadsheets (U.K.: *M* = 11.04; *SD* = 26.770; CH: *M* = 2.95; *SD* = 11.657). Moreover, as Table 2 shows, in the U.K., tabloids (*M* = 16.35; *SD* = 30.234) allocated also more attention to business misinformation than rival broadsheets (*M* = 7.92; *SD* = 13.971). In Switzerland, however, the tabloid (*M* = 1.40; *SD* = 4.395) allocated less attention to business misinformation than the corresponding rival broadsheet (*M* = 1.89; *SD* = 6.166).

#### Moderators

The first moderator refers to the media market. In the U.K. (= 1), news media have faced higher expected costs due to press councils’ sanctions [43, 44] and higher competition [53, 54]. In Switzerland (= 0), in turn, news media have faced lower expected costs due to the press council’s sanctions [42] and lower competition [53, 54].

The second moderator refers to the ideological utility of misinformation. Drawing on Kepplinger, Brosius and Staab [57], this was measured as follows: The misinformation has ideological benefits for a news outlet (= 1) if it undermines actors and issues that relate to contrary political and economic positions or if it supports actors and issues that relate to shared positions. Conversely, the misinformation has ideological costs for a news outlet (= 0) if it supports actors and issues that relate to contrary political and economic positions or if it undermines actors and issues that relate to shared positions.

To assess the ideological utility, in a first step, the political and economic alignments of actors and issues were determined, which were subject of the misinformation: conservative vs. social liberal (political misinformation); free market vs. market regulation (business misinformation). For instance, it was investigated whether a conservative or a left-wing political party was subject of political misinformation and whether a company or a regulator was subject of business misinformation. Moreover, it was investigated whether the misinformation undermined or supported these actors and issues. This was done based on an analysis of press councils’ rulings as well as based on desk research.

In a second step, the misinformation was compared with broadsheets’ editorial lines: towards conservatism and free-market orientation (i.e., *Neue Zürcher Zeitung*, *The Daily Telegraph* and *The Times*) vs. towards social liberalism and regulated market orientation (i.e., *Tages-Anzeiger* and *The Guardian*). Editorial lines were determined based on the *eurotopics* database (which is administered by the Federal Agency for Civic Education in Germany) as well as based on desk research. After all, particularly in the U.K., broadsheets adapted their support for political parties throughout the investigated time frame.

As Table 1 shows, the investigated U.K. and Swiss cases of political misinformation had on average nearly the same ideological utility for U.K. (*M* = .58; *SD* = .497) and Swiss (*M* = .59; *SD* = .501) broadsheets, respectively. This holds also for business misinformation (Table 2): U.K. (*M* = .48; *SD* = .503); Switzerland (*M* = .51; *SD* = .504).

#### Controls

Drawing on herd behavior literature, further factors were controlled. First, actors are more likely to engage in imitation if the first mover is an expert who is assumed to have (more) reliable information [50, 51]. Accordingly, two types of news outlets were differentiated, which first published the political or business misinformation: the first mover is a supraregional up-market news outlet (= 1); the first mover is another type of news outlet (e.g., tabloid, regional or local news outlet) (= 0). The expert status of the first movers was determined via press councils’ rulings.

As Table 1 shows, in the U.K. (*M* = .39; *SD* = .490), first movers, which broke political misinformation, were more likely supraregional up-market news outlets than in Switzerland (*M* = .17; *SD* = .384). This holds also for first movers, which broke business misinformation (Table 2): U.K. (*M* = .30; *SD* = .462); Switzerland (*M* = .21; *SD* = .413).

Moreover, actors are more likely to engage in imitation the stronger the first mover’s signal is [49]. Therefore, the news value of political and business misinformation was controlled. Drawing on Kepplinger and Ehmig [58] as well as fög [37], the news value was measured based on the following news factors, which were weighted and summed up into news value indices: national status (1 = local; 3 = regional; 9 = national); hierarchical status (1 = lower level; 2 = middle level; 3 = higher level); personalization (0 = no personalization; 1 = professional life; 2 = private life; 3 = intersection of professional and private life); prominence (0 = not prominent personalized actor; 3 = prominent personalized actor); intimacy (0 = intimate issue of personalized actor is not addressed; 3 = intimate issue of personalized actor is addressed); relevance (1 = consequences for one individual; 2 = consequences for two or more individuals; 3 = consequences for one organization; 4 = consequences for two or more organizations; 5 = consequences for one system; 6 = consequences for two or more systems); negativity (1 = positive; 2 = neutral; 3 = negative). The news values were determined via press councils’ rulings.

As Table 1 shows, in the U.K. (*M* = 19.18; *SD* = 4.279), political misinformation had on average a higher news value than in Switzerland (*M* = 16.14; *SD* = 4.373). This holds also for business misinformation (Table 2): U.K. (*M* = 21.36; *SD* = 4.948); Switzerland (*M* = 18.95; *SD* = 4.322).

Furthermore, since news media’s susceptibility to engage in imitation might increase over time as media markets get more disrupted and newsroom resources are increasingly cut [59], the year in which the political and business misinformation broke was controlled (2002 = 2; 2018 = 18). The years were determined via press councils’ rulings.

As Table 1 shows, in the U.K. (*M* = 13.55; *SD* = 4.381), political misinformation broke on average during later years of the investigated time frame than in Switzerland (*M* = 9.00; *SD* = 4.870). This holds also for business misinformation (Table 2): U.K. (*M* = 14.27; *SD* = 4.341); Switzerland (*M* = 9.59; *SD* = 4.978). This suggests that, over time, U.K. news outlets published more misinformation and/or U.K. press councils processed and upheld more accuracy violations.

Finally, contrary to the Swiss press council [42], U.K. press councils have regulated only newspapers with a member status [43, 44]. While *The Guardian* was a member of the *Press Complaints Commission* (= 1), the newspaper didn’t join the *Independent Press Standards Organization* in 2014 and was subsequently coded with = 0. Swiss broadsheets (*Neue Zürcher Zeitung* and *Tages-Anzeiger*) and the other U.K. broadsheets (*The Daily Telegraph* and *The Times*) were coded with = 1 for the whole investigated time frame.

Accordingly, in the U.K., a smaller percentage of broadsheets were regulated than in Switzerland. This holds for political journalism (U.K.: *M* = .77; *SD* = .423; CH: *M* = 1.00; *SD* = .000) (Table 1) and for business journalism (U.K.: *M* = .75; *SD* = .434; CH: *M* = 1.00; *SD* = .000) (Table 2).

The descriptive statistics are summarized in Tables 1 and 2. The bivariate correlations are indicated in Tables 3 and 4.

**Table 3:** Bivariate correlations (political journalism).

	1	2	3	4	5	6	7	8	9
Election campaigns	—								
Media market: U.K. (ref. = CH)	.044	—							
Attention to misinformation: RB	.338**	.133	—						
Attention to misinformation: TA	.324**	.153	.609**	—					
Ideological utility: IB (ref. = IC)	.053	-.009	.016	.107	—				
First mover: HS (ref. = LS)	.294**	.184*	.188*	.022	-.009	—			
Misinformation: News value	.147	.276**	.249**	.340**	.011	.076	—		
Misinformation: Year	-.138	.381**	-.150	-.004	-.035	-.109	-.077	—	
Broadsheets: RE (ref. = NR)	-.055	-.240**	.022	.018	.259**	-.001	-.027	-.378**	—
Attention to misinformation: BR	.172*	.011	.369**	.605**	.211*	.000	.235**	-.140	.124

*p < .05

**p < .01. N = 142.

U.K. = English part of the U.K.; CH = German-speaking part of Switzerland; RB = rival broadsheets; TA = tabloids; IB = ideological benefits; IC = ideological costs; HS = higher expert status; LS = lower expert status; RE = regulated by press council; NR = not regulated by press council; BR = broadsheets.

**Table 4:** Bivariate correlations (business journalism).

	1	2	3	4	5	6	7	8	9
Economic downturns	—								
Media market: U.K. (ref. = CH)	.259**	—							
Attention to misinformation: RB	.141	.263**	—						
Attention to misinformation: TA	.210*	.316**	.451**	—					
Ideological utility: IB (ref. = IC)	-.018	-.029	.018	.115	—				
First mover: HS (ref. = LS)	-.171*	.100	.207*	-.139	-.110	—			
Misinformation: News value	.204*	.250**	.276**	.431**	.049	.120	—		
Misinformation: Year	.330**	.452**	.192*	.148	.008	-.148	.113	—	
Broadsheets: RE (ref. = NR)	-.103	-.360**	-.210*	-.094	.038	-.065	-.148	-.349**	—
Attention to misinformation: BR	.071	.038	.241**	.212*	.058	.152	.169	.092	.071

*p < .05

**p < .01. N = 134.

U.K. = English part of the U.K.; CH = German-speaking part of Switzerland; RB = rival broadsheets; TA = tabloids; IB = ideological benefits; IC = ideological costs; HS = higher expert status; LS = lower expert status; RE = regulated by press council; NR = not regulated by press council; BR = broadsheets.

### Data analysis

To test hypotheses 1a-b and 3a-b, linear regressions were performed with SPSS. Moreover, to test hypotheses 2a-b and 4a-b, moderation analyses were performed with the PROCESS macro for SPSS [60]. While the first three controls (expert status, news value and year) were used for all statistical analyses, the fourth control (regulation), which relates to individual broadsheets, was only used for the tests of hypotheses 3a-b and 4a-b.

Furthermore, the statistical significance of the moderated mediations was examined with the PROCESS macro for SPSS [60]. More specifically, in the case of political journalism, a moderated parallel mediation model was chosen, which included both mediators as well as the second moderator (ideological utility). As the statistical analysis revealed that the first moderator (media market) has no significant effects, it was not included in the model. In the case of business journalism, in turn, a dual moderated parallel mediation model was chosen. It included both mediators as well as both moderators (media market and ideological utility). Statistical significance was tested using 10,000 bootstrapped samples to estimate 95% bias-corrected confidence intervals. A moderated mediation is significant when the 95% confidence interval doesn't include zero [60].

## Findings

As Table 5 shows, election campaigns are significantly and positively related to tabloids’ (*β* = .313, *p* = < .001) as well as rival broadsheets’ (*β* = .273, *p* = .001) attention to political misinformation. In fact, as the coefficients indicate, tabloids allocated more attention to political misinformation the closer election dates approached than rival broadsheets. Accordingly, H1a is supported. However, as Table 6 shows, economic downturns have neither a significant effect on tabloids’ (*β* = .047, *p* = .566) nor on rival broadsheets’ (*β* = .063, *p* = .478) attention to business misinformation–at least across both media markets. H1b is, therefore, not supported.

**Table 5:** Linear regressions and moderation analyses (political journalism).

Mediator variable models: Rival broadsheets’ attention to misinformation						
	Model 1		Model 2			
	Β	P	B	SE	t	p
Election campaigns	.273	.001	1.220	.369	3.305	.001
Media market: U.K. (ref. = CH)	.118	.199	8.341	5.714	1.460	.147
Election campaigns x Media market	—	—	.927	.994	.932	.353
First mover: HS (ref. = LS)	.059	.485	1.733	4.543	.381	.704
Misinformation: News value	.161	.055	.810	.468	1.732	.086
Misinformation: Year	-.139	.117	-.770	.453	-1.698	.092
Constant	—	—	-6.009	11.874	-.506	.614
Model	R^2^ = .150; p < .001. N = 142.		R^2^ = .185; F(6, 135) = 5.113; p < .001. N = 142.			
Mediator variable models: Tabloids’ attention to misinformation						
	Model 1		Model 2			
	Β	P	B	SE	t	p
Election campaigns	.313	< .001	1.530	.399	3.840	< .001
Media market: U.K. (ref. = CH)	.070	.437	4.931	6.169	.799	.425
Election campaigns x Media market	—	—	.201	1.074	.187	.852
First mover: HS (ref. = LS)	-.102	.219	-6.022	4.905	-1.228	.222
Misinformation: News value	.284	.001	1.700	.505	3.365	.001
Misinformation: Year	.024	.785	.118	.489	.242	.809
Constant	—	—	-30.979	12.820	-2.416	.017
Model	R^2^ = .177; p < .001. N = 142.		R^2^ = .206; F(6, 135) = 5.849; p < .001. N = 142.			
Dependent variable models: Broadsheets’ attention to misinformation						
	Model 1		Model 2			
	Β	P	B	SE	t	p
Election campaigns	-.043	.573	-.134	.134	-.997	.320
Attention to misinformation: RB	-.008	.928	.000	.035	.009	.993
Attention to misinformation: TA	.598	< .001	.189	.034	5.606	< .001
Ideological utility: IB (ref. = IC)	.137	.052	3.502	1.372	2.552	.012
RB x Ideological utility	—	—	-.055	.067	-.829	.409
TA x Ideological utility	—	—	.270	.067	4.006	< .001
First mover: HS (ref. = LS)	-.015	.838	.399	1.460	.273	.785
Misinformation: News value	.031	.669	.098	.155	.628	.531
Misinformation: Year	-.129	.087	-.255	.150	-1.698	.092
Broadsheets: RE (ref. = NR)	.028	.712	-.058	1.898	-.030	.976
Constant	—	—	4.045	4.321	.936	.351
Model	R^2^ = .374; p < .001. N = 142.		R^2^ = .484; F(10, 131) = 12.298; p < .001. N = 142.			

U.K. = English part of the U.K.; CH = German-speaking part of Switzerland; HS = higher expert status; LS = lower expert status; RB = rival broadsheets; TA = tabloids; IB = ideological benefits; IC = ideological costs; RE = regulated by press council; NR = not regulated by press council.

**Table 6:** Linear regressions and moderation analyses (business journalism).

Mediator variable models: Rival broadsheets’ attention to misinformation						
	Model 1		Model 2			
	Β	P	B	SE	t	p
Economic downturns	.063	.478	.745	.668	1.115	.267
Media market: U.K. (ref. = CH)	.121	.205	2.422	2.230	1.086	.279
Economic downturns x Media market	—	—	1.041	1.206	.863	.390
First mover: HS (ref. = LS)	.201	.020	5.189	2.222	2.335	.021
Misinformation: News value	.195	.024	.428	.209	2.053	.042
Misinformation: Year	.124	.194	.262	.212	1.236	.219
Constant	—	—	-13.566	5.053	-2.685	.008
Model	R^2^ = .127; p < .001. N = 134.		R^2^ = .165; F(6, 127) = 4.181; p = .001. N = 134.			
Mediator variable models: Tabloids’ attention to misinformation						
	Model 1		Model 2			
	Β	P	B	SE	t	p
Economic downturns	.047	.566	4.576	1.196	3.825	< .001
Media market: U.K. (ref. = CH)	.253	.005	8.767	3.993	2.195	.030
Economic downturns x Media market	—	—	9.178	2.160	4.249	< .001
First mover: HS (ref. = LS)	-.211	.009	-11.686	3.980	-2.936	.004
Misinformation: News value	.390	< .001	1.591	.374	4.260	< .001
Misinformation: Year	-.057	.518	-.383	.379	-1.008	.315
Constant	—	—	-27.681	9.049	-3.059	.003
Model	R^2^ = .250; p < .001. N = 134.		R^2^ = .368; F(6, 127) = 12.339; p < .001. N = 134.			
Dependent variable models: Broadsheets’ attention to misinformation						
	Model 1		Model 2			
	Β	P	B	SE	t	p
Economic downturns	.020	.825	.009	.198	.043	.966
Attention to misinformation: RB	.136	.182	.145	.054	2.704	.008
Attention to misinformation: TA	.140	.189	-.008	.028	-.286	.775
Ideological utility: IB (ref. = IC)	.049	.568	.726	.971	.748	.456
RB x Ideological utility	—	—	-.368	.100	-3.682	< .001
TA x Ideological utility	—	—	.177	.053	3.355	.001
First mover: HS (ref. = LS)	.174	.065	1.486	1.238	1.201	.232
Misinformation: News value	.056	.560	.069	.115	.602	.548
Misinformation: Year	.119	.218	.100	.107	.942	.348
Broadsheets: RE (ref. = NR)	.174	.058	1.830	1.599	1.144	.255
Constant	—	—	-2.769	3.309	-.837	.404
Model	R^2^ = .070; p = .028. N = 134.		R^2^ = 0.229; F(10, 123) = 3.649; p < .001. N = 134.			

U.K. = English part of the U.K.; CH = German-speaking part of Switzerland; HS = higher expert status; LS = lower expert status; RB = rival broadsheets; TA = tabloids; IB = ideological benefits; IC = ideological costs; RE = regulated by press council; NR = not regulated by press council.

Moreover, the media market doesn’t significantly moderate the relationships between election campaigns and tabloids’ (*B* = .201, *t*(135) = .187, *p* = .852) as well as rival broadsheets’ (*B* = .927, *t*(135) = .932, *p* = .353) attention to political misinformation (Table 5). Therefore, during election campaigns, tabloids and rival broadsheets in the U.K. didn’t allocate more attention to political misinformation than tabloids and rival broadsheets in Switzerland. Accordingly, H2a is not supported.

In fact, the media market doesn’t significantly moderate the relationship between economic downturns and rival broadsheets’ attention to business misinformation either (*B* = 1.041, *t*(127) = .863, *p* = .390) (Table 6). Therefore, in the case of rival broadsheets, H2b is not supported. In the case of tabloids, however, the findings reveal a significant difference. They show that tabloids in the U.K. allocated more attention to business misinformation the stronger economic downturns became than tabloids in Switzerland (*B* = 9.178, *t*(127) = 4.249, *p* < .001). Accordingly, in the case of tabloids, H2b is supported.

Moreover, while tabloids’ attention to political misinformation is significantly and positively related to broadsheets’ attention to political misinformation (*β* = .598, *p* < .001), rival broadsheets’ attention to political misinformation has no significant effect (*β* = -.008, *p* = .928) (Table 5). Accordingly, H3a is supported. However, in the case of business journalism (Table 6), neither tabloids’ (*β* = .140, *p* = .189) nor rival broadsheets’ (*β* = .136, *p* = .182) attention to misinformation has a significant effect–at least across both levels of ideological utility. H3b is, therefore, not supported.

Furthermore, the ideological utility of political misinformation significantly and positively moderates the relationship between tabloids’ and broadsheets’ attention to political misinformation (*B* = .270, *t*(131) = 4.006, *p* < .001) (Table 5). However, the ideological utility doesn’t significantly moderate the relationship between rival broadsheets’ and broadsheets’ attention to political misinformation (*B* = -.055, *t*(131) = -.829, *p* = .409). Accordingly, in the case of tabloids, H4a is supported.

In the case of business journalism (Table 6), in turn, the ideological utility significantly and positively moderates the relationship between tabloids’ and broadsheets’ attention to misinformation (*B* = .177, *t*(123) = 3.355, *p* = .001), while it significantly and negatively moderates the relationship between rival broadsheets’ and broadsheets’ attention to misinformation (*B* = -.368, *t*(123) = -3.682, *p* < .001). Therefore, in the case of tabloids, H4b is supported.

Finally, in the case of tabloids’ attention to political misinformation (Table 7), the moderated mediation is significant (*Index* = .414, 95% CI = .044 to .907). This confirms that broadsheets allocated more attention to political misinformation during election campaigns the more attention tabloids allocated to the misinformation and if the misinformation had ideological benefits. This holds for broadsheets in the U.K. as well as for broadsheets in Switzerland. However, in the case of rival broadsheets’ attention to political misinformation, the moderated mediation is not significant (*Index* = -.068, 95% CI = -.458 to .251).

**Table 7:** Moderated parallel mediation analysis (political journalism).

Index of moderated mediation: Rival broadsheets’ attention to misinformation				
	Index	SE	CI LL	CI UL
	-.068	.175	-.458	.251
Indices of conditional mediation: Rival broadsheets’ attention to misinformation				
	Index	SE	CI LL	CI UL
Misinformation with ideological costs	.039	.068	-.090	.183
Misinformation with ideological benefits	-.028	.159	-.356	.308
Index of moderated mediation: Tabloids’ attention to misinformation				
	Index	SE	CI LL	CI UL
	.414	.224	.044	.907
Indices of conditional mediation: Tabloids’ attention to misinformation				
	Index	SE	CI LL	CI UL
Misinformation with ideological costs	.051	.054	-.022	.187
Misinformation with ideological benefits	.464	.234	.078	.978
Direct effect				
	B	SE	t	p
Election campaigns	-.134	.134	-.997	.320

In fact, the findings indicate the same attention allocation process for business journalism. After all, in the case of tabloids’ attention to business misinformation (Table 8), the moderated moderated mediation is significant (*Index* = 1.626, 95% CI = .101 to 3.655). This confirms that broadsheets allocated more attention to business misinformation during economic downturns the more attention tabloids allocated to the misinformation and if the misinformation had ideological benefits. However, this holds for broadsheets in the U.K. media market in particular. In the case of rival broadsheets’ attention to business misinformation, in turn, the moderated moderated mediation is not significant (*Index* = -.383, 95% CI = -1.704 to .778).

**Table 8:** Dual moderated parallel mediation analysis (business journalism).

Index of moderated moderated mediation: Rival broadsheets’ attention to misinformation				
	Index	SE	CI LL	CI UL
	-.383	.592	-1.704	.778
Indices of conditional moderated mediation: Rival broadsheets’ attention to misinformation				
	Index	SE	CI LL	CI UL
Swiss media market	-.065	.078	-.253	.053
U.K. media market	-.449	.585	-1.750	.660
Index of moderated moderated mediation: Tabloids’ attention to misinformation				
	Index	SE	CI LL	CI UL
	1.626	.913	.101	3.655
Indices of conditional moderated mediation: Tabloids’ attention to misinformation				
	Index	SE	CI LL	CI UL
Swiss media market	-.075	.073	-.251	.027
U.K. media market	1.551	.873	.096	3.521
Direct effect				
	B	SE	t	p
Economic downturns	.009	.198	.043	.966

**Fig 2:**
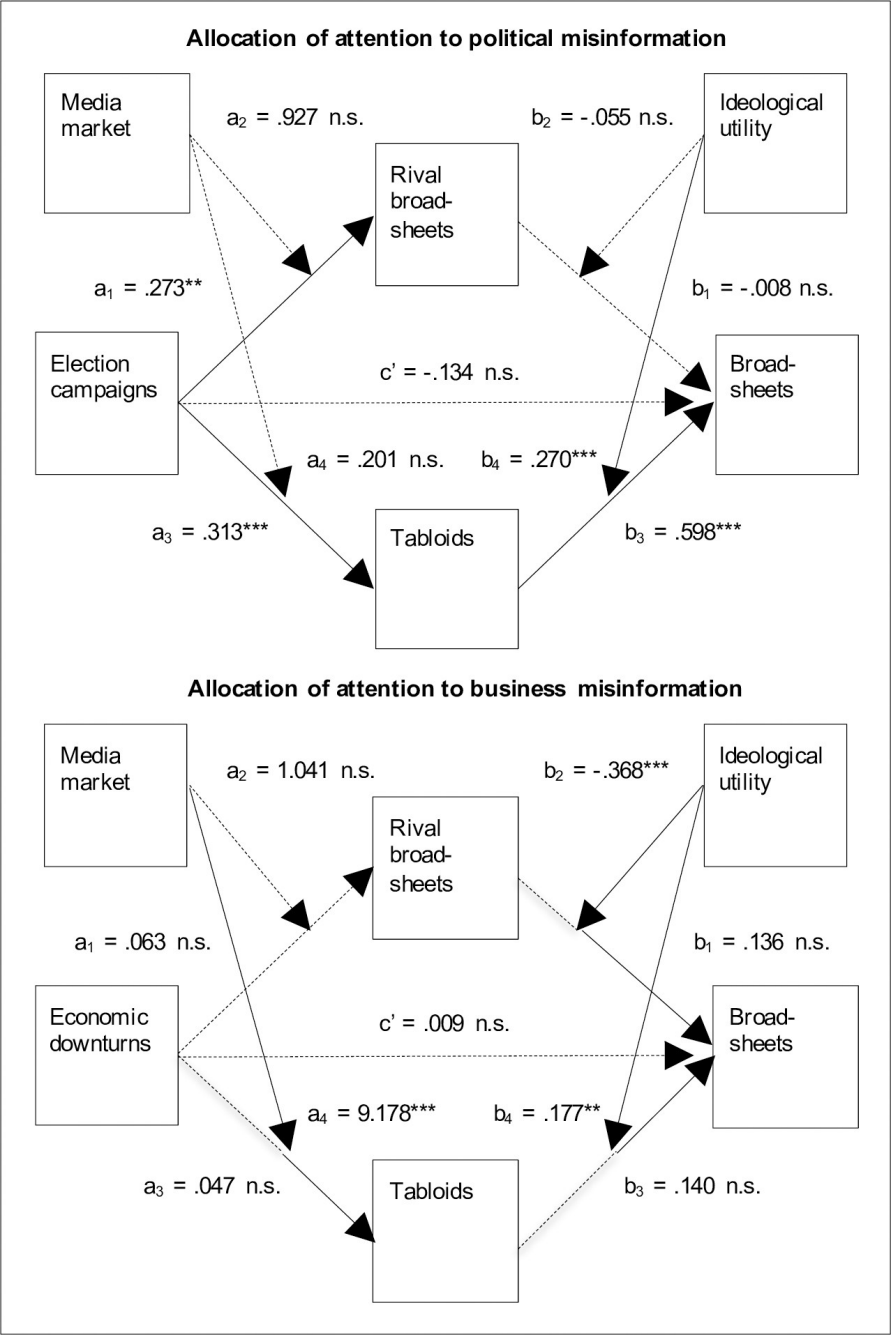
Summary of the findings.

Furthermore, election campaigns (*B* = -.134, *p* = .320) and economic downturns (*B* = .009, *p* = .966) have no significant direct effects on broadsheets’ attention to political and business misinformation, respectively (Tables 7 and 8). Accordingly, as summarized in Fig 2, tabloids’ attention to political and business misinformation fully mediates the relationships between election campaigns and economic downturns as well as broadsheets’ attention to political and business misinformation, respectively. However, this holds particularly under specific conditions: ideological benefits (political journalism); U.K. media market and ideological benefits (business journalism).

## Discussion

This study developed and tested a theoretical framework, which draws on herd behavior literature and explains how and under what conditions tabloids’ attention to misinformation drives broadsheets’ attention to misinformation. Moreover, the study applied a replication logic and examined the theoretical framework in two news ecosystems, i.e., in political and business journalism. In fact, the findings reveal an attention allocation process, which occurs in both news ecosystems and, therefore, indicate that this attention allocation process is a broader phenomenon in journalism.

More specifically, the findings show that if misinformation enters a media market during an amplifying event–i.e., if a first-mover news outlet publishes political or business misinformation during election campaigns or economic downturns, respectively–tabloids (i.e., newspapers with a mass- or mid-market orientation) in particular allocate increased attention to the political or business misinformation. This, in turn, drives broadsheets (i.e., newspapers with an up-market orientation) to allocate increased attention to the misinformation as well.

Interestingly, rival broadsheets’ increased attention to political or business misinformation has no positive effect. This indicates that–during amplifying events, which intensify competition in media markets,–broadsheets (as collective actors) are more likely influenced by the availability heuristic [6] than by reputational [47] or informational [49] incentives.

After all, actors are more susceptible to join reputational and informational cascades if they consist of experts who are assumed to have (more) reliable information [50, 51, 61]. This is because following such experts enables actors to reduce reputational damage should a decision turn out to be wrong (i.e., they can share the blame with experts) and to reduce costs of information search (i.e., they can follow supposedly accurate signals of experts). Therefore, if broadsheets had reputational or informational incentives, they would have rather followed rival broadsheets.

Availability cascades, in turn, work differently. Actors imitate others and adopt information because they take the simple availability of information as an indication of its reliability and relevance [6]. In fact, the findings show that as election dates approached and economic downturns became stronger, tabloids allocated more attention to political and business misinformation, respectively, than rival broadsheets. Thereby, tabloids made the misinformation more publicly available and, accordingly, had a greater influence [62] on broadsheets’ decisions on how to allocate attention. In that sense, the availability heuristic [6] might have a similar effect as the bandwagon heuristic [63], which refers to the tendency of actors to engage in collective behavior that has become popular, and which has been applied in previous research to investigate the diffusion of (mis-)information [64, 65].

Moreover, as Kuran and Sunstein point out, “the availability heuristic can produce systematic and persistent misperceptions” ([6] page 690), which, in turn, might have negative consequences for society. In fact, while Mullainathan and Shleifer suggest that pressure from rivals “forces news outlets to seek and deliver more accurate information” ([66] page 1031), the findings support Sutter’s argument that in times of increased rivalry–i.e., during amplifying events–“[f]als information gets much greater coverage […] than otherwise” ([20] page 747) and flows from tabloids to broadsheets.

In fact, broadsheets follow tabloids not blindly but rather strategically. After all, the findings show that tabloids’ attention to misinformation drives broadsheets’ attention to misinformation particularly under the condition that the misinformation serves broadsheets’ ideological goals [52], i.e., if it undermines actors and issues, which relate to contrary political and economic positions, or if it supports actors and issues, which relate to shared political and economic positions. This doesn’t necessarily mean that broadsheets know that the information is inaccurate [7]; it is also possible that they are unaware that the information is inaccurate or that they are uncertain whether it is correct. Yet, this suggests that broadsheets report the information nevertheless without sufficient verification–due to increased competition during amplifying events [20] and expected ideological benefits [52].

However, this attention allocation process is not generally more pronounced in a media market, which consists of more news media and, therefore, is characterized by higher competition. After all, the findings show differences between Swiss and U.K. media markets only in the case of business journalism but not in the case of political journalism. While it was assumed that higher expected benefits due to stronger competition in a media market (U.K.) will outweigh higher expected costs due to stronger press councils’ sanctions (U.K) and, therefore, will drive U.K. news media to allocate more attention to misinformation than Swiss news media (i.e., lower competition and weaker sanctions), the findings suggest that higher expected costs might balance out higher expected benefits.

The differences between U.K. and Swiss media markets in the case of business journalism might, instead, be rather attributed to different strengths of the amplifying events. After all, the GDP per capita rates indicate that in the U.K., economic downturns were more pronounced than in Switzerland (Table 2). This suggests that the attention allocation process depends in particular on the strength of the amplifying event in a media market. More specifically, the findings indicate that the stronger an amplifying event is, the more attention will tabloids allocate to corresponding misinformation, which, in turn, will drive broadsheets to a higher extent to allocate more attention to the misinformation as well–and especially if the misinformation serves broadsheets’ ideological goals.

## Conclusions

While previous research has focused on how inaccurate information spreads on social media, the diffusion of misinformation in news media has received less attention and is, therefore, less well understood. However, as misinformation poses risks for societies and news media shape public opinion [3, 7], a deeper understanding of underlying mechanisms is crucial to be better able to contain the spread of misinformation in journalism.

Therefore, this article contributes to the understanding of how and under what conditions misinformation diffuses in media markets. The findings show that during amplifying events (i.e., election campaigns and economic downturns) tabloids allocate more attention to political and business misinformation, which, in turn, drives broadsheets to allocate more attention to the misinformation as well–and especially if the misinformation serves broadsheets’ ideological goals. Moreover, the findings suggest that this attention allocation process depends in particular on the strength of the amplifying event in a media market.

Thereby, the findings further challenge the notion that broadsheets are particularly important opinion leaders among traditional news media and set the news agenda of other types of news media (e.g., [14–18]). After all, the findings suggest that during amplifying events, which increase competition in media markets, tabloids assume the role of opinion leaders and shape broadsheets’ decisions on how to allocate attention. This is concerning, as broadsheets are expected to act as “bouncers of the public sphere and truth’s keeper[s]” ([11] page xi) and, accordingly, are considered to be particularly relevant for the functioning of societies [12].

Moreover, while it is in broadsheets’ self-interest to assure the accuracy of news [67, 68], media accountability [69] and media governance [70] instruments might need to play an increasingly important role in the future. Press councils have been revising their workflows and codes of practice to become more efficient and effective in the digital age. However, “media accountability and media governance […] must be seen as a process of different but interrelated practices […]” ([71] page 334). In fact, a higher diversity of actors who are involved in media accountability and media governance activities–for instance, different types of fact-checkers [72–74]–might facilitate the containment of misinformation in journalism [75].

Several limitations of this study need to be addressed. First, the study reveals significant relationships between tabloids’ attention to political and business misinformation as well as broadsheets’ attention to political and business misinformation, respectively. However, causality has still to be established (e.g., [17]). Therefore, a promising path for future research would be to investigate these relationships under controlled conditions, i.e., based on experiments (which could also consider micro-level influences).

Second, the study investigated two media markets–namely Switzerland and the U.K. To further examine how the spread of misinformation differs depending on the media market, future research might compare other countries, which differ in terms of media regulation (i.e., with vs. without a press council; stronger vs. weaker press council’s sanctions) and market structure (i.e., higher vs. lower competition).

Third, the study selected the investigated cases of political and business misinformation based on press councils’ rulings, which upheld accuracy violations. This allowed defining and investigating specific populations, which, however, don’t incorporate all misinformation, which emerged in the analyzed media markets during the examined time frame. Moreover, the findings reveal a distinct attention allocation process. However, they show also that the number of print articles in which the investigated Swiss and U.K. news media adopted misinformation is limited; or not as extensive as it might be intuitively assumed–towards the “‘post-truth’ era” ([2] page 353).

Accordingly, a further path for future research would be to examine the detected attention allocation process in other contexts–for instance in online journalism. More specifically, future research might investigate how interactions between tabloids’ and broadsheets’ news websites play out in the spread of misinformation. Due to the restricted access to online articles, such studies might investigate more recent time frames and, instead, compare a broader sample of media markets. After all, shortening production cycles and increasing time pressures in newsrooms might rather intensify the allocation of attention to misinformation.

## References

[1] Allcott H, Gentzkow M. Social media and fake news in the 2016 election. Journal of Economic Perspectives. 2017;31(2):211–236.

[2] Lewandowsky S, Ecker UKH, Cook J. Beyond misinformation: Understanding and coping with the “post-truth” era. Journal of Applied Research in Memory and Cognition. 2017;6(4):353–369.

[3] Russ-Mohl S. Die informierte Gesellschaft und ihre Feinde. Warum die Digitalisierung unsere Demokratie gefährdet [The Informed Society and Its Enemies. Why the Digitalization Puts Our Democracy at Risk]. Köln: Herbert von Halem Verlag; 2017.

[4] Bennett WL, Livingston S. The disinformation order: Disruptive communication and the decline of democratic institutions. European Journal of Communication. 2018;33(2):122–139.

[5] Humprecht E, Esser F, van Aelst P. Resilience to online disinformation: A framework for cross-national comparative research. The International Journal of Press/Politics. 2020;25(3):493–516.

[6] Kuran T, Sunstein CR. Availability cascades and risk regulation. Stanford Law Review. 1999;51(4):683–768.

[7] Ha L, Perez LA, Ray R. Mapping recent development in scholarship on fake news and misinformation, 2008 to 2017: Disciplinary contribution, topics, and impact. American Behavioral Scientist. 2019; online first: https://doi.org/10.1177%2F0002764219869402. 31693016

[8] Wardle C, Derakhshan H. Information Disorder: Toward an Interdisciplinary Framework for Research and Policy Making. Strasbourg: Council of Europe; 2017.

[9] Tandoc EC, Lim ZW, Ling R. Defining “fake news”. A typology of scholarly definitions. Digital Journalism. 2018;6(2):137–153.

[10] Doyle G. Understanding Media Economics. London: SAGE; 2013.

[11] Hendricks VF, Vestergaard M. Reality Lost: Markets of Attention, Misinformation and Manipulation. Springer Open; 2019.

[12] Hamilton JT. Democracy’s Detectives: The Economics of Investigative Journalism. Cambridge, MA: Harvard University Press; 2016.

[13] Guo L, Vargo C. “Fake news” and emerging online media ecosystem: An integrated intermedia agenda-setting analysis of the 2016 U.S. presidential election. Communication Research. 2020;47(2):178–200.

[14] Mathes R, Pfetsch B. The role of the alternative press in the agenda-building process: Spill-over effects and media opinion leadership. European Journal of Communication. 1991;6(1):33–62.

[15] Shoemaker P, Reese SD. Mediating the Message. New York, NY: Routledge; 2011.

[16] Golan G. Inter-media agenda setting and global news coverage: Assessing the influence of the New York Times on three network television evening news programs. Journalism Studies. 2006;7(2):323–333.

[17] Vonbun R, Kleinen-von Königslöw K, Schönbach K. Intermedia agenda-setting in a multimedia news environment. Journalism. 2016;17(8):1054–1073.

[18] Mathis T, umprecht E. Werden Leitmedien häufiger zitiert? Eine empirische Untersuchung von Schweizer Printmedien [Are leading media more often quoted? An empirical study of Swiss print media]. Medien & Kommunikationswissenschaft [Media & Communication Science]. 2018;66(1):41–57.

[19] Downs A. Up and down with ecology–The “issue-attention cycle”. Public Interest. 1972;28(1):38–50.

[20] Sutter D. The social costs of media feeding frenzies. International Journal of Social Economics. 2001;28(9):742–751.

[21] Del Vicario M, Bessi A, Zollo F, Petroni F, Scala A, Caldarelli G, et al The spreading of misinformation online. PNAS. 2016;113(3):554–559. 10.1073/pnas.1517441113 26729863 PMC4725489

[22] Allcott H, Gentzkow M, Yu C. Trends in the diffusion of misinformation on social media. Research & Politics. 2019;6(2):1–8.

[23] Burger P, Kanhai S, Pleijter A, Verberne S. The reach of commercially motivated junk news on Facebook. PLoS ONE. 2019;14(8): e0220446 10.1371/journal.pone.0220446 31369596 PMC6675076

[24] Grinberg N, Joseph K, Friedland L, Swire-Thompson B, Lazer D. Fake news on Twitter during the 2016 U.S. presidential election. Science. 2019;363(6425):374–378. 10.1126/science.aau2706 30679368

[25] Cinelli M, Cresci S, Galeazzi A, Quattrociocchi W, Tesconi M. The limited reach of fake news on Twitter during 2019 European elections. PLoS ONE. 2020;15(6): e0234689 10.1371/journal.pone.0234689 32555659 PMC7302448

[26] Zhao Z, Zhao J, Sano Y, Levy O, Takayasu H, Takayasu M, et al Fake news propagates differently from real news even at early stages of spreading. EPJ Data Science. 2020;9(7): 10.1140/epjds/s13688-020-00224-z.

[27] Silverman C. Lies, Damn Lies, and Viral Content. How News Websites Spread (and Debunk) Online Rumors, Unverified Claims, and Misinformation. Tow Center for Digital Journalism. Columbia Journalism School; 2015.

[28] Vargo CJ, Guo L, Amazeen MA. The agenda-setting power of fake news: A big data analysis of the online media landscape from 2014 to 2016. New Media & Society. 2018;20(5):2028–2049.

[29] Banerjee AV. A simple model of herd behavior. The Quarterly Journal of Economics. 1992;107(3):797–817.

[30] Bikhchandani S, Sharma S. Herd Behavior in Financial Markets: A Review. IMF Working Paper. Washington: International Money Fund; 2000.

[31] Ho B, Liu P. Herd journalism: Investment in novelty and popularity in markets for news. Information Economics and Policy. 2015;31(June):33–46.

[32] Wilczek B. Herd behavior and path dependence in news markets. Towards an economic theory of scandal formation. Journal of Interdisciplinary Economics. 2016;28(2):137–167.

[33] Chung IJ. Dynamics of media hype: Interactivity of the media and the public. In: Vasterman P, editor. From Media Hype to Twitter Storm. News Explosions and Their Impact on Issues, Crises and Public Opinion. Amsterdam: Amsterdam University Press; 2018 p. 211–228.

[34] Hameleers M, Bos L, de Vreese CH. Shoot the messenger? The media’s role in framing populist attributions of blame. Journalism. 2019;20(9):1145–1164.

[35] Knowles S, Phillips G, Lidberg J. Reporting the global financial crisis: A longitudinal tri-nation study of mainstream financial journalism. Journalism Studies. 2017;18(3):322–340.

[36] Quiring O, Kepplinger HM, Weber M, Geiss S. Lehman Brothers und die Folgen: Berichterstattung zu wirtschaftlichen Interventionen des Staates [Lehman Brothers and the Consequences: News Coverage about State Interventions]. Wiesbaden: Springer VS; 2013.

[37] fög (Forschungszentrum Öffentlichkeit und Gesellschaft), editor. Jahrbuch Qualität der Medien [Yearbook Media Quality]. Basel: Schwabe Verlag; 2019.

[38] Diekmann A, Przepiorka W, Rauhut H. Lifting the veil of ignorance: An experiment on the contagiousness of norm violations. Rationality and Society. 2015;27(3):309–333.

[39] Cohen-Almagor R. After Leveson: Recommendations for instituting the public and press council. The International Journal of Press/Politics. 2014;19(2):202–225.

[40] Puppis M. Organisationen der Medienselbstregulierung: Europäische Presseräte im Vergleich [Organizations for Media Self-Regulation: A Comparison of European Press Councils]. Köln: Herbert von Halem Verlag; 2009.

[41] Fielden L. Regulating the Press: A Comparative Study of International Press Councils. Reuters Institute for the Study of Journalism. University of Oxford; 2012.

[42] Schweizer Presserat. Erklärung der Pflichten und Rechte der Journalistinnen und Journalisten [Declaration of Duties and Rights of Journalists]. Bern; 2018.

[43] PCC (Press Complaints Commission). Editors’ Code of Practice. London; 2014.

[44] IPSO (Independent Press Standards Organization). Editors’ Code of Practice. London; 2018.

[45] Lacy S, Martin HJ. Competition, circulation and advertising. Newspaper Research Journal. 2004;25(1):18–39.

[46] Fletcher R, Park S. The impact of trust in the news media on online news consumption and participation. Digital Journalism. 2017;5(10):1281–1299.

[47] Scharfstein DS, Stein JC. Herd behavior and investment. The American Economic Review. 1990;80(3):465–479.

[48] Hamilton JT. All the News That’s Fit to Sell: How the Market Transforms Information into News. Princeton: Princeton University Press; 2004.

[49] Bikhchandani S, Hirshleifer D, Welch I. A theory of fads, fashion, custom, and cultural change as informational cascades. Journal of Political Economy. 1992;100(5):992–1026.

[50] Lemieux P. Following the herd. Regulation. 2003–2004;26(4):16–21.

[51] Hedström P. Rational imitation. In: Hedström P, Swedberg R, editors. Social Mechanisms: An Analytical Approach to Social Theory. Cambridge: Cambridge University Press; 1998 p. 306–328.

[52] Tandoc EC. The facts of fake news: A research review. Sociology Compass. 2019;13(9):1–9.

[53] Hallin DC, Mancini P. Comparing Media Systems: Three Models of Media and Politics. Cambridge: Cambridge University Press; 2004.

[54] Picard RG, Russi L. Comparing media markets. In: Esser F, Hanitzsch T, editors. The Handbook of Comparative Communication Research. London: Routledge; 2012 p. 234–248.

[55] Lacy S, Watson BR, Riffe D, Lovejoy J. Issues and best practices in content analysis. Journalism & Mass Communication Quarterly. 2015;92(4):791–811.

[56] Neuendorf KA. The Content Analysis Guidebook. Thousand Oaks, Calif: SAGE; 2017.

[57] Kepplinger HM, Brosius HB, Staab JF. Instrumental actualization: A theory of mediated conflicts. European Journal of Communication. 1991;6(3):263–290.

[58] Kepplinger HM, Ehmig SC. Predicting news decisions: An empirical test of the two-component theory of news selection. Communications. 2006;31(1):25–43.

[59] Wilczek B. Complexity, uncertainty and change in news organizations: Towards a cycle model of digital transformation. The International Journal on Media Management. 2019;21(2):88–129.

[60] Hayes AF. Introduction to Mediation, Moderation, and Conditional Process Analysis. A Regression-Based Approach. 2nd ed. New York: Guilford Press; 2018.

[61] Keuschnigg M. Imitation und Konformität [imitation and conformity]. In: Braun N, Saam NJ, editors. Handbuch Modellbildung und Simulation in den Sozialwissenschaften [The Handbook of Model Building and Simulation in the Social Sciences]. Wiesbaden: Springer VS; 2015. p. 903–934.

[62] Frank RH. Under the influence: Putting peer pressure to work. Princeton: Princeton University Press; 2020.

[63] Leibenstein H. Bandwagon, snob, and veblen effects in the theory of consumers’ demand. The Quarterly Journal of Economics. 1950;64(2):183–207.

[64] Törnberg P. Echo chambers and viral misinformation: Modeling fake news as complex contagion. PLoS ONE. 2018;13(9): e0203958 10.1371/journal.pone.0203958 30235239 PMC6147442

[65] Wang C-J, Zhu JJH. Jumping onto the bandwagon of collective gatekeepers: Testing the bandwagon effect of information diffusion on social news website. Telematics and Informatics. 2019;41(August):34–45.

[66] Mullainathan S, Shleifer A. The market for news. The American Economic Review. 2005;95(4):1031–1053.

[67] Kalogeropoulos A, Suiter J, Udris L, Eisenegger M. News media trust and news consumption: Factors related to trust in news in 35 countries. International Journal of Communication. 2019;13:3672–3693.

[68] Fletcher R, Nielsen RK. Paying for online news. A comparative analysis of six countries. Digital Journalism. 2017;5(9):1173–1191.

[69] Fengler S, Eberwein T, Leppik-Bork T. Mapping media accountability–in Europe and beyond. In: Eberwein T, Fengler S, Lauk E, Leppik-Bork T, editors. Mapping Media Accountability–in Europe and Beyond. Köln: Herbert von Halem Verlag; 2011 p. 7–21.

[70] Puppis M. Media governance as a horizontal extension of media regulation: The importance of self- and co-regulation. Communications. 2007;32(3):330–336.

[71] Eberwein T, Porlezza C. Both sides of the story: Communication ethics in mediatized worlds. Journal of Communication. 2016;66(2):328–342.

[72] Graves L. Boundaries not drawn. Mapping the institutional roots of the global fact-checking movement. Journalism Studies. 2018;19(5):613–631.

[73] Shao C, Hui P-M, Wang L, Jiang X, Flammini A, Menczer F, et al Anatomy of an online misinformation network. PLoS ONE. 2018;13(4): e0196087 10.1371/journal.pone.0196087 29702657 PMC5922526

[74] Pingree RJ, Watson B, Sui M, Searles K, Kalmoe NP, Darr JP, et al Checking facts and fighting back: Why journalists should defend their profession. PLoS ONE. 2018;13(12): e0208600 10.1371/journal.pone.0208600 30532136 PMC6287821

[75] Southwell BG, Thorson EA. The prevalence, consequences, and remedy of misinformation in mass media systems. Journal of Communication. 2015;65(4):589–595.

